# A high-speed railway network dataset from train operation records and weather data

**DOI:** 10.1038/s41597-022-01349-8

**Published:** 2022-05-27

**Authors:** Dalin Zhang, Yunjuan Peng, Yi Xu, Chenyue Du, Yumei Zhang, Nan Wang, Yunhao Chong, Hongwei Wang, Daohua Wu, Jintao Liu, Hailong Zhang, Lingyun Lu, Jiqiang Liu

**Affiliations:** 1 School of Software Engineering, Beijing Jiaotong University, Beijing, 100044 China; 2 National Research Center of Railway Safety Assessment, Beijing Jiaotong University, Beijing, 100044 China; 3 Department of Computer and Information Science, Fordham University, New York City, 10458 USA

**Keywords:** Scientific data, Decision making, Geography

## Abstract

High-speed train operation data are reliable and rich resources in data-driven research. However, the data released by railway companies are poorly organized and not comprehensive enough to be applied directly and effectively. A public high-speed railway network dataset suitable for research is still lacking. To support the research in large-scale complex network, complex dynamic system and intelligent transportation, we develop a high-speed railway network dataset, containing the train operation data in different directions from October 8, 2019 to January 27, 2020, the train delay data of the railway stations, the junction stations data, and the mileage data of adjacent stations. In the dataset, weather, temperature, wind power and major holidays are considered as factors affecting train operation. Potential research values of the dataset include but are not limited to complex dynamic system pattern mining, community detection and discovery, and train delay analysis. Besides, the dataset can be used to solve various railway operation and management problems, such as passenger service network improvement, train real-time dispatching and intelligent driving assistance.

## Abstract (summary)

**Table:** 

Measurement(s)	train operation data of China high-speed railway network • locations of railway stations • weather and temperature and wind power
Technology Type(s)	web scraping with python • Geographic Information System
Factor Type(s)	high-speed train departure delay time • high-speed train arrival delay time • various external factors affecting train operation • bad weather exposure time of railway stations • railway station community size
Sample Characteristic - Organism	high-speed train
Sample Characteristic - Environment	railway system
Sample Characteristic - Location	China

## Background & Summary

Since the 21st century, high-speed railway has developed rapidly. A large number of train operation data have been generated. At present, data-driven modeling method are widely used in intelligent transportation and other fields. Train operation data have been considered as rich and reliable resources in data modeling research^1^, which mainly include the railway lines, schedule and actual operation time of the train, and occupancy and release time of the track, etc. However, the acquisition of the train operation data is still a big challenge. The data generated by train operation are collected and saved by railway companies. Due to the lack of organization, it is difficult to be used directly. In addition, researchers often need to construct available data sets by simulating, measuring or combining the data released by different institutions because the internal and external environmental information of train operation cannot be effectively provided by the companies. To the best of the authors’ knowledge, no public available high-speed railway network dataset suitable for research has been reported.

The complexity of high-speed railway network is significant, which mainly includes the following aspects: (1) The train operation status of different time and lines have distribution characteristics^2–4^ and spatio-temporal correlation^5–7^. In the spatial dimension, the train operation rules of different stations and lines are different, and there are spatial interactions between trains. For example, some stations are more likely to delay due to geographical location and climate. In the temporal dimension, the train operation status has temporal trend and dependence characteristics. For example, the peak of delay often occurs in a few months of each year. (2) The high-speed railway network in the actual operation scenario is dynamic^8^. In different time, due to the dynamic change of the train lines, the adjacency relationships between stations are different, and the structure of the constructed graph is also different. This dynamic includes the constantly changing characteristics of stations, lines, and the network structure (the appearance or the disappearance of new lines and stations). (3) The high-speed railway network has dynamic community structure^4^. According to the dynamic of the network, the types of stations, and the multiple characteristics of the operating lines, the high-speed railway network can be divided into multiple dynamic communities, and the stations within the same community follow the similar train operating rules. (4) Train operations are affected by various external factors^3,4,7,9^, such as weather and unexpected events. The bad external environment is easy to cause abnormal operation of the train from different extents. We will show these complex characteristics of high-speed railway network in detail in the subsequent Data Records as well as Technical Validation sections.

In summary, it is critical to share and publish the multi-attribute high-speed railway network dataset with real-world distributions, not only for optimizing transportation organization, but also helpful to model various structures of the network.

In this paper, we create a unique high-speed railway network dataset that covers 3,399 high-speed trains and 727 railway stations in China. The dataset contains the train operation data in different directions from October 8, 2019 to January 27, 2020, the number of delays at the stations in different periods and directions, the data of main junction stations, and the mileage data of adjacent stations on the train diagram. Weather, temperature, wind power and major holidays are considered as factors affecting train operation in the dataset to make it more valuable for research.

The high-speed railway network dataset can be processed as the materials for effective methods to issue the problems in large-scale complex network, complex dynamical system, intelligent transportation, deep learning, data mining and other fields, including but not limited to complex network modeling^10–12^, complex dynamic system pattern mining^5,13–15^, travel demand analysis^16^, community detection and discovery^17–19^, urban accessibility research^20,21^, train delay analysis^6,7,22–24^, task mining on multi-scale and dynamic graphs^25–27^. In addition, it can be used to optimize the actual railway operation and management, such as (a) train operation scheme and schedule adjustment, (b) passenger service network improvement, (c) train speed, punctuality, capacity, and energy consumption prediction, (d) real-time dispatching, (e) intelligent driving assistance, (f) fault or accident detection and (g) maintenance plans making.

## Methods

To obtain the high-speed railway network dataset, we first collect the train operation records, mileage information and the geographical locations of the railway stations. The historical weather related data are collected based on the geographical locations, and the dates of major holidays from October 8, 2019 to January 27, 2020 are obtained. Second, we calculate the arrival and departure delay time of one train and count the number of delayed trains per hour in different directions of one station. Third, compute the mileage of adjacent stations. Fourth, train operation conditions of China’s top ten junctions are statistics. Fifth, according to the geographical locations and time stamps, the train directions, station types, weather, holidays and other complex factors are expanded to the operation data of high-speed trains and delay data of railway stations. Finally, we check and validate our dataset.

**Fig. 1:**
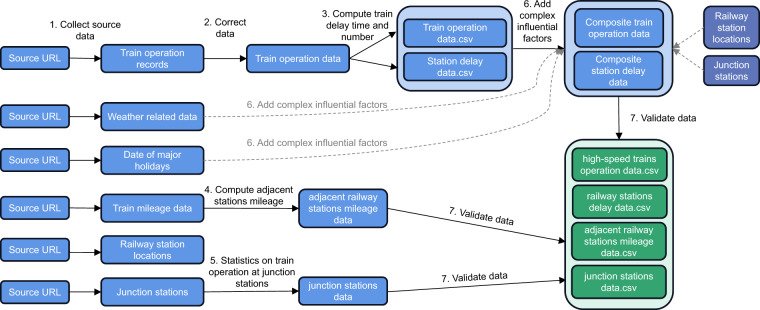
Flow chart of methodology. The figure shows the flowchart of methodology to obtain the high-speed railway network dataset from train operation records and weather data.

Figure 1 shows the flowchart of methodology to obtain the high-speed railway network dataset from train operation records and weather data. The steps involved are described in detail below.

### Step 1. Source data collection

The source data for the high-speed railway network dataset consists of the high-speed trains operation data, the high-speed trains mileage data, the locations of railway stations, the junction stations, the weather related data and the major holidays.

#### High-speed train operation records collection

**Fig. 2:**
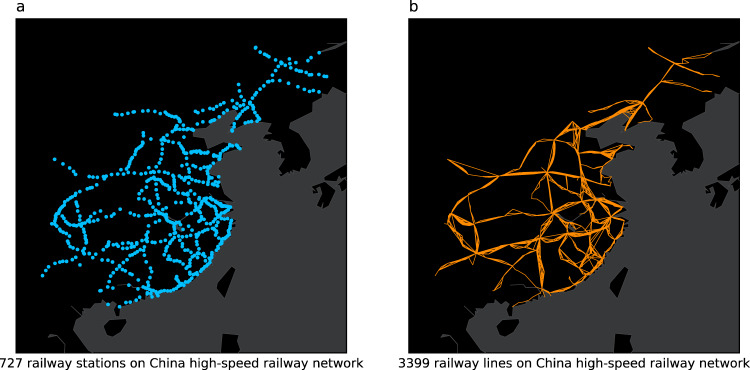
China high-speed railway network. The figure shows the actual operation network of China Railway High Speed, which includes the 727 railway stations and 3,399 high-speed trains in the dataset. (**a**) shows location of stations. (**b**) shows the railway lines.

High-speed train operation records consist of the historical schedule and actual operation information. We use the web scraping method with python^28^ to obtain 2,751,713 running data of 3,399 trains from China Railway Ticket System (https://www.12306.cn), from October 8, 2019 to January 27, 2020, 16 weeks in total. The operation records of one train consist of stopping stations, scheduled departure and arrival time, actual departure and arrival time, etc. Fig. 2 shows China high-speed railway network, the 727 stations and actual operation lines of 3,399 trains are included.

#### High-speed trains mileage data collection

According to the train operation records, we use the web scraping method to obtain the operating mileage of 3,399 trains from http://www.huochepiao.com. We obtain the data updated to 2020 because the railway routes are constantly adjusted. The attributes contained in the data include train number, station order, station name and the mileage between one station and the departure station. We supplement the missing mileage data by manual search.

#### Locations of railway stations collection

We get 727 stations after deleting the duplicates based on the 3,399 high-speed trains operating lines. The names of these stations are unique. Then, we get the geographic locations of them, which include the province, city and district. We supplement the missing location information by manual search.

#### Junction stations collection

In the railway network, the connection place of several trunk lines is generally called railway junction, which is composed of several stations, inter-station connecting lines, inbound lines and signals. In the dataset, we consider ten representative junctions in China, the stations are shown in Table 1.

**Table 1:** Junction stations.

Junction	Station name
Beijing	Beijingbei Railway Station
	Beijingnan Railway Station
	Beijingxi Railway Station
	Qinghe Railway Station
Zhengzhou	Zhengzhou Railway Station
	Zhengzhoudong Railway Station
	Zhengzhouxi Railway Station
Wuhan	Hankou Railway Station
	Wuhan Railway Station
	Tianhejichang Railway Station
Shanghai	Shanghai Railway Station
	Shanghaihongqiao Railway Station
	Shanghaixi Railway Station
	Nanxiangbei Railway Station
Guangzhou	Guangzhoubei Railway Station
	Guangzhounan Railway Station
	Qingsheng Railway Station
Xian	Xianbei Railway Station
	Afanggong Railway Station
Shenyang	Shenyang Railway Station
	Shenyangbei Railway Station
	Shenyangnan Railway Station
Nanjing	Nanjing Railway Station
	Nanjingnan Railway Station
	Jiangning Railway Station
	Jiangningxi Railway Station
	Xianlin Railway Station
Guiyang	Guiyangdong Railway Station
	Guiyangbei Railway Station
Changsha	Changshanan Railway Station
	Zhuzhouxi Railway Station
	Xiangtanbei Railway Station

#### Weather related data collection

It is reported that the operation of high-speed train is affected by climate, such as strong wind, low temperature and torrential rain. So we consider weather, wind power, and temperature as external influential factors to make the dataset more valuable for research. We crawl the data for 16 weeks from a website (http://www.tianqihoubao.com) that records historical weather related data by matching the districts where the stations located in. The data contains a total of 81,242 weather related samples from 727 districts.

We use the Scrapy-Redis multi-task asynchronous framework to crawl the above data and store them in MongoDB database. To improve the efficiency of I/O operations, we use mongoexport to store the data in a csv file.

#### Major holidays collection

It is well known that the passenger flow is also an important factor influencing train operation. When multiple trains are late, dispatchers often need to decide the train departure order based on the capacity and the real number of passengers of one station. However, we can not accurately obtain the real number of passengers at one station due to the high mobility of passengers. Luckily, it is clear that the number of passengers tends to be higher than usual during the holidays, especially major holidays, such as Spring Festival and National Day. Therefore, we take major holidays as one of the external influencing factors.

From October 8, 2019 to January 27, 2020, the major holidays considered are Halloween on October 31, 2019, Thanksgiving Day on November 28, 2019, National Memorial Day on December 13, 2019, Christmas on December 25, 2019, New Year’s Day on January 1, 2020, Laba Festival on January 2, 2020, Chinese New Year’s Eve on January 24, 2020, and Spring Festival on January 25, 2020.

### Step 2. Data Correction

In this step we correct the collected high-speed train operation records. There are some missing and wrong information in the records, which will affect the computation of train delay time and delay number. Therefore, it is crucial to correct the records before judging and computing delayed trains.

To prevent the loss of observations that may be valuable, we fill in the missing values with data close to them on the date. That is because, for one train, its running status shows a certain trend, which generally remains consistent in the same period.

In the process of data collection, we find that the actual departure time is smaller than the actual arrival time in some of the operation records, which is impossible in the real train operation scene. We regard them as abnormal data. In most cases, one train runs normally according to the schedule, and the stop time at one station is also planned. Therefore, we compute the sum of actual arrival time and scheduled stop time to replace the abnormal actual departure time.

### Step 3. Delay time and number computation

In this step, we compute the delay time of one train on its operation line and count the number of delayed trains per hour in different directions at one station. The delay of one high-speed train includes departure delay and arrival delay. So we mainly construct these two attributes in our dataset.

#### Delay time computation for high-speed trains

The original collected high-speed train operation records contains the train running dates, the name of the stations passing by the trains, station order, scheduled departure time and arrival time, actual departure time and arrival time, stop time, etc.

For one station *S*, the schedule defines that one train should arrive at time ${t}_{A}^{S}$ and leave at time ${t}_{D}^{S}$ after stopping at station *S* for a period of time. In most cases, the schedule is accurate, which means that most trains will depart and arrive on time. However, due to uncontrollable reasons such as extreme weather and large passenger flow, trains may not depart or arrive on time. The actual arrival and departure time are defined as ${\widehat{t}}_{A}^{S}$ and ${\widehat{t}}_{D}^{S}$. Then ${\widehat{t}}_{A}^{S}-{t}_{A}^{S}$ is defined as arrive not on time, ${\widehat{t}}_{D}^{S}-{t}_{D}^{S}$ is defined as depart not on time. Apparently, when ${\widehat{t}}_{A}^{S}-{t}_{A}^{S} > 0$, it shows that the train arrives late at *S*; ${\widehat{t}}_{D}^{S}-{t}_{D}^{S} > 0$ shows that the train departs late at *S*. When ${\widehat{t}}_{A}^{S}-{t}_{A}^{S} < 0$, it shows that the train arrives at *S* ahead of time; ${\widehat{t}}_{D}^{S}-{t}_{D}^{S} < 0$ shows that the train departs at *S* ahead of time.

According to the above definition, we add attributes “departure delay” and “arrival delay” in the high-speed train operation data. We compute the time of non-on-time arrive and depart. When these two values are bigger than 0, they represent the time of train delays. when these two values are smaller than 0, they represent the time of train departs or arrives early. It is worth noting that one train has no arrival delay at the departure station, so the value of “arrival delay” is always 0, and no departure delay at the terminal station, so the value of “departure delay” is always 0. We store the final processing results in a csv file.

#### Delay number computation for railway stations

The departure time of one train depends on the scheduling strategy of one station when the delay occurs. Analyzing the number of historical train delays at one station and mining the existing rules can help railway dispatching. It is also an effective way to evaluate the dispatching capacity of one station. In a word, statistic on the number of arrival and departure delayed trains at one station is very valuable.

The operation line of one train is directional, which is divided into up and down. According to China Railway, “up” means that the train is leaving for Beijing or running from the branch line to the trunk line (the train number is even number), “down” means that the train is leaving to Beijing or running from the trunk line to the branch line (the train number is odd number). From [00:00, 01:00), October 8, 2019 to [23:00, 24:00), January 27, 2020, we take one hour as a time step to compute the number of departure delays and arrival delays at 727 stations. Supposing that the train number of one train passing through station *S* is *T*, the number of trains with $T=2\times n$ is *U*, the number of trains with $T=2\times (n-1)$ is *W*, then the number of arrival delayed trains in the upward direction is $\mathop{\sum }\limits_{i=1}^{U}\;\left({\widehat{t}}_{A}^{S}-{t}_{A}^{S} > 0\right)$, in downward direction is $\mathop{\sum }\limits_{i=1}^{W}\;\left({\widehat{t}}_{A}^{S}-{t}_{A}^{S} > 0\right)$, the number of departure delayed trains in upward direction is $\mathop{\sum }\limits_{i=1}^{U}\;\left({\widehat{t}}_{D}^{S}-{t}_{D}^{S} > 0\right)$, in downward direction is $\mathop{\sum }\limits_{i=1}^{W}\;\left({\widehat{t}}_{D}^{S}-{t}_{D}^{S} > 0\right)$. We store the delay number data of the railway stations in a csv file.

### Step 4. Adjacent stations mileage computation

In the high-speed railway network dataset, adjacent stations refer to neighboring stations on the train diagram that are not geographically close to each other (separated by multiple small stations). Since the lines in different directions between two adjacent stations may be different, resulting in different distances between them, we add direction attribute to the mileage data of adjacent stations (high-speed railway network is a directed network). That is, we calculate the mileage between adjacent stations in the upward and downward directions. According to the high-speed trains mileage data, we can get the distance ${M}_{{S}_{i}}$ between one station ${S}_{i}$ and departure station, and then the distance between adjacent stations is ${M}_{{S}_{i}}-{M}_{{S}_{i-1}}$.

### Step 5. Train operation at junction stations statistics

In this step, we compute the total number of the upward and downward trains, the upward and downward arrival delayed trains and departure delayed trains passing through each junction station from October 8, 2019 to January 27, 2020. The above data can be easily computed by matching Table 1 and the junction station names in the high-speed train operation data.

### Step 6. Complex influential factors adding

In this step, we need to add the train direction, station type, weather related data and major holidays to the processed train operation data and delay number data of railway stations.

#### Train direction and station type adding

The direction of one train is divided into upward and downward. By judging whether the train number is odd or even, we get the operation direction and combine it with the train operation data. Station types include junction stations and non junction stations. By matching the station names in Table 1 and delay number data of railway stations, we can easily judge whether one station is a junction station and combine it with the station delay data.

#### Weather related data adding

Weather, wind power and temperature information of 727 stations in 16 weeks are contained in the weather related data. By matching the dates and station names, we obtain the train operation data and delay data of stations with weather related factors.

#### Major holidays adding

The major holidays are on October 31, 2019, November 28, 2019, December 13, 2019, December 25, 2019, January 1, 2020, January 2, 2020, January 24, 2020 and January 25, 2020. We respectively add the attribute “holiday” to the train operation data and the delay data of stations. The value of “holiday” is “True” or “False”. By matching dates, we judge whether the dates in the train operation data and the delay data of stations are included in the above 8 dates.

Through the above data processing methods, we obtain the final high-speed railway network dataset.

### Step 7. Data validation

We perform validation steps for the high-speed railway network dataset from train operation records and weather data. Please see Section “Technical Validation” for more details.

## Data Records

### Complexity of high-speed railway network dataset

Considering the influence of network structure, weather and other factors on train operation, our high-speed railway network dataset is complex. To fully mining the potential value of the dataset, it is necessary to establish complex learning models, such as graph convolution neural network.

The complexity of our high-speed railway network dataset shows in: (1) the temporal and spatial distribution characteristics of train operation; (2) dynamic of high-speed railway network; (3) dynamic community of high-speed railway network; (4) the diversity of external influencing factors of train operation.

Specifically, the dataset contains corresponding attributes to model these complex characteristics, such as station type (junction stations are more likely to affect other stations), train operation direction (different lines in different directions affect different areas), length of the railway lines (from the perspective of delay, the longer the distance, the greater the possibility of delay recovery), weather, temperature, wind level and major holidays (factors affecting train operation).

#### Temporal and spatial distribution characteristics

**Fig. 3:**
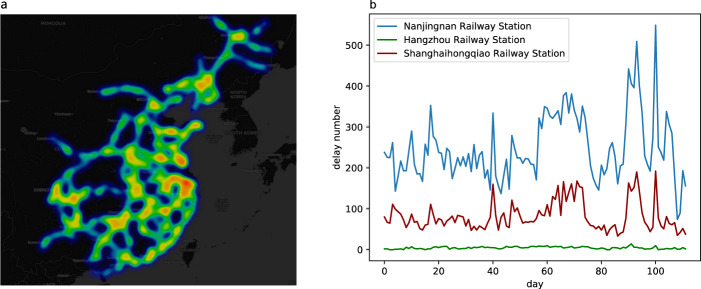
Spatio-temporal distribution of station delay. (**a**) shows the heat map of spatial distribution of station delay. (**b**) shows the temporal distribution of train delay.

Taking the total number of delays at stations as an example, we draw the temporal and spatial distribution of station delays, as shown in Fig. 3. In spatial dimension, stations with a large number of delays are concentrated in Shanghai, Nanjing, Shenyang and other areas, which contain multiple junction stations (Fig. 3a). When the train passing through these stations is delayed, it will lead to the delay of other trains in multiple directions and lines, and the delay propagation is more serious than that of other stations. In temporal dimension, we take three stations as an example to draw the figure of train delay number from October 8, 2019 to January 27, 2020 (Fig. 3b). There was almost no delay at Hangzhou Railway station, while the number of train delays at Nanjingnan Railway Station and Shanghaihongqiao Railway station showed a continuous peak in December. In addition, the delay at the stations is consistent with the historical delay, and the stations that have been delayed in the past are more likely to be delayed in the future.

#### Dynamic characteristic

**Fig. 4:**
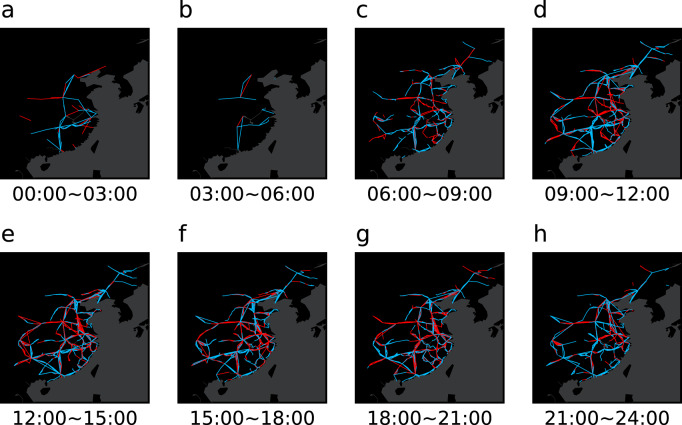
Dynamic of high-speed railway network. (**a**)-(**h**) respectively show the train network structure in different time. The red lines indicate the delayed railway lines, and the blue lines indicate the normal railway lines.

According to the high-speed train operation scheme, the operation lines of one train will change continuously in one day (the operation line here refers to the line between two stations). Taking January 16, 2020 as an example, we draw the dynamic operation network in Fig. 4. The blue lines represent the railway lines in normal operation, and the red lines represent the railway lines in delay. Few trains operated from 00:00 to 06:00. However, trains run through almost all stations on the network in other time. Compared with other time, the delay of trains from 09:00 to 21:00 was more serious, which indicates that the train delay network is also dynamic.

#### Dynamic community characteristic

**Fig. 5:**
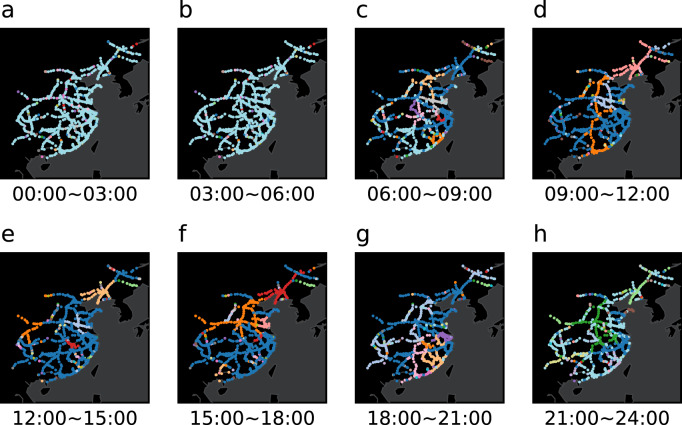
Dynamic community of high-speed railway network. (**a**)-(**h**) respectively show the community structure of train operation network in different time. Among them, stations of same color belong to the same community.

On the dynamic high-speed railway network, stations can be divided into multiple communities. Stations belonging to the same community often obey similar train operation rules. On the basis of Fig. 4, we draw the train dynamic community network based on Louvain algorithm^29^, as shown in Fig. 5. Different colors in the figure represent different communities. Because few trains running from 00:00 to 06:00, most stations had no trains passing through, so they are divided into the same community. According to the location of stations, changing train operation lines, changing delay status and etc., the community structure of train operation network is also constantly changing.

### Data records description

This dataset^30^ is located in figshare, which is available as 4 separate csv files described as follows:

- high-speed trains operation data.csv: the operation data of 3,399 high-speed trains from October 8, 2019 to January 27, 2020 with major holidays and weather related influencing factors.
- railway stations delay data.csv: number of delayed trains at 727 railway stations from [00:00, 01:00), October 8, 2019 to [23:00, 24:00), January 27, 2020 with major holidays and weather related influencing factors.
- adjacent railway stations mileage data.csv: mileage data of adjacent stations on 3,399 train operation lines.
- junction stations data.csv: data of China’s top ten junctions, including the total number of trains and delayed trains passing through one station in different directions from October 8, 2019 to January 27, 2020.

The relevant fields of these files are listed out in Tables 2 to 5.

**Table 2:** High-speed trains operation data.

Column	Data type	Description
date	date	The operating dates of the high-speed trains.
train_number	string	The number of the high-speed trains.
train_direction	string	Operation directions of the high-speed trains.
station_name	string	The names of the railway stations that the high-speed trains passe through.
station_order	integer	The order of the railway stations that the high-speed trains pass through.
scheduled_arrival_time	time	The scheduled arrival time of the high-speed trains.
scheduled_departure_time	time	The scheduled departure time of the high-speed trains.
stop_time	integer	The scheduled stop time of the high-speed trains at the stations (unit: minute).
actual_arrival_time	time	The actual arrival time of the high-speed trains.
actual_departure_time	time	The actual departure time of the high-speed trains.
arrival_delay	integer	The arrival delayed time of the high-speed trains (unit: minute).
departure_delay	integer	The departure delayed time of the high-speed trains (unit: minute).
wind	string	Wind power on the operating days in the area of the stations.
weather	string	Weather on the operating days in the area of the stations.
temperature	integer	Temperature on the operating days in the areas of the stations. (unit: centigrade).
major_holiday	bool	Whether one operating day is a major holiday (the value is “True” or “False”).

**Table 3:** Railway stations delay data.

Column	Data type	Description
station_name	string	The names of the stations.
station_type	string	Whether one station is a junction station.
province	string	The province where one station is located in.
city	string	The city where one station is located in.
district	string	The district where one station is located in.
start_time	timestamp	Time to start recording the number of delayed high-speed trains.
end_time	timestamp	Time to stop recording the number of delayed high-speed trains.
up_arrival_delay_number	integer	Train arrival delays in the upward direction from start time to end time.
down_arrival_delay_number	integer	Train arrival delays in the downward direction from start time to end time.
up_departure_delay_number	integer	Train departure delays in the upward direction from start time to end time.
down_departure_delay_number	integer	Train departure delays in the downward direction from start time to end time.
wind	string	Wind power on the operating days in the areas of the stations.
weather	string	Weather on the operating days in the areas of the stations.
temperature	integer	Temperature on the operating days in the areas of the stations. (unit: centigrade).
major_holiday	bool	Whether one operating day is a major holiday (the value is “True” or “False”).

**Table 4:** Adjacent stations mileage data.

Column	Data type	Description
from_station	string	Departure station of one train.
to_station	string	Arrival station of one train.
mileage	integer	Mileage between one departure station and one arrival station.
train_direction	string	Operation direction of one high-speed train.

We consider the adjacent stations on the train diagram.

**Table 5:** Junction stations data.

Column	Data type	Description
station_name	string	The name of the stations.
province	string	Province where one station is located.
city	string	City where one station is located.
district	string	District where one station is located.
up_number	integer	The number of upward trains passing through one station.
down_number	integer	The number of downward trains passing through one station.
up_arrival_delay_number	integer	The number of train arrival delays in the upward direction.
down_arrival_delay_number	integer	The number of train arrival delays in the downward direction.
up_departure_delay_number	integer	The number of train departure delays in the upward direction.
down_departure_delay_number	integer	The number of train departure delays in the downward direction.

We only consider the stations located in China’s top ten junctions.

## Technical Validation

This section is to validate if the high-speed railway network dataset from train operation records and weather data can reflect the real operation of trains. We validate the dataset by integrating numerical comparison and disciplinary analysis from the following four aspects.

- The correctness of the train operating diagram.
- The distribution characteristics of train operation.
- Correlation between train operation and external influencing factors.
- Power-law distribution characteristic of railway station community.

### High-speed train operation diagram check

To validate the correctness of the scheduled operation data in the high-speed trains operation data.csv, we obtain the 2019 and 2020 train schedules from http://www.gaotie.cn/. We validate the consistency by comparing the scheduled departure time and arrival time, scheduled stop time, the name and order of the stations passing by the trains and other information of the trains. From October 8, 2019 to January 27, 2020, there were a total of 2,751,713 trains, 27,092 trains leaving from Beijingnan Railway Station, 45,391 trains leaving from Guangzhounan Railway Station, and 208 trains leaving from Yimianpobei Railway Station.

### Validation on distributions of train operation

Train operation has the distribution characteristics of the actual running time and stop time^2^. To further validate the reliability of our dataset, we validate these two characteristics.

#### The relationship between train operation status and actual running time

Due to the low running speed before one train enters one station, the train that arrives on time or ahead of time have more redundant time in the last block range, which makes it quite different from the operation scheme of the delayed trains. Therefore, the running time of one train in one section is affected by the delay of the departure stations.

**Fig. 6:**
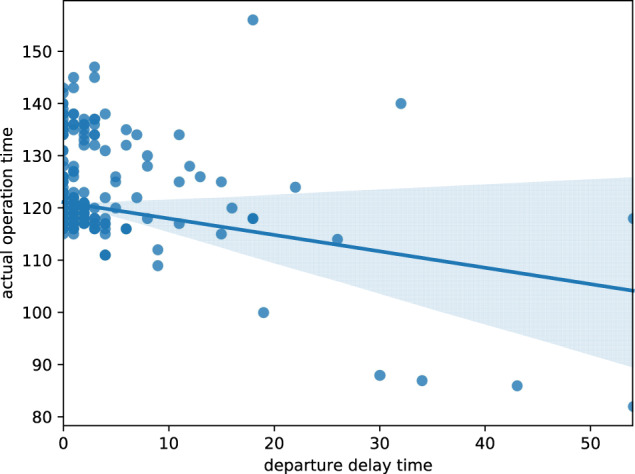
Actual running time distribution characteristic of high-speed train operation. The figure shows the correlation between departure delay time at Jinanxi Railway Station and actual running time from Jinanxi Railway Station to Nanjingnan Railway Station.

Taking Jinanxi Railway Station to Nanjingnan Railway Station as an example, we choose the departure time at Jinanxi Railway Station and the actual operation time from Jinanxi Railway Station to Nanjingnan Railway Station to analyze the relationship between the actual running time and train operation status. Taking the departure delay time as the horizontal coordinate and the actual operation time as the vertical coordinate, a scatter plot is drawn and a fitted curve is generated as shown in Fig. 6. As the departure delay time at Jinanxi Railway Station increases, the actual running time from Jinanxi Railway Station to Nanjingnan Railway Station gradually decreases. This is consistent with the research conclusion of literature 2.

#### The relationship between train operation status and actual stop time

**Fig. 7:**
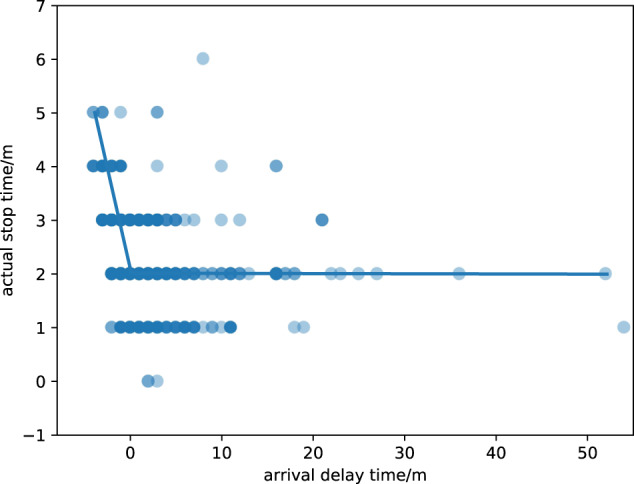
Actual stop time distribution characteristic of high-speed train operation. The figure shows the correlation between arrival delay time and actual stop time.

To explore the relationship between train operation status and actual stop time, we choose the train operation time from Jinanxi Railway Station to Nanjingnan Railway Station and the actual stop time at Nanjingnan Railway Station for analysis. When the scheduled stop time at Nanjingnan is 2 minutes, we take the arrival delay time at Nanjingnan as the horizontal coordinate, and the actual stop time at Nanjingnan as the vertical coordinate, a scatter plot is drawn and a fitted curve is generated as shown in Fig. 7. When the arrival delay time is smaller than or equal to 0, the actual stop time decreases with the increase of the arrival delay time because the train can not depart ahead of time; when the arrival delay time is bigger than 0, with the increase of the arrival delay time, the actual stop time of the train gradually decreases. Because the actual stop time is bigger than or equal to the minimum actual stop time, the reduction range of the actual stop time is also gradually decreasing, and finally approaches the minimum stop time and keeps stable. This is also consistent with the research conclusion of literature 2.

### Validation on correlation between high-speed train operation and external factors

External environmental factors significantly affect the operation of the train. In order to verify the availability and reliability of weather related data in our dataset, we validate it from the relationship between train operation and external factors.

#### The relationship between high-speed train delay rate and external factors

**Fig. 8:**
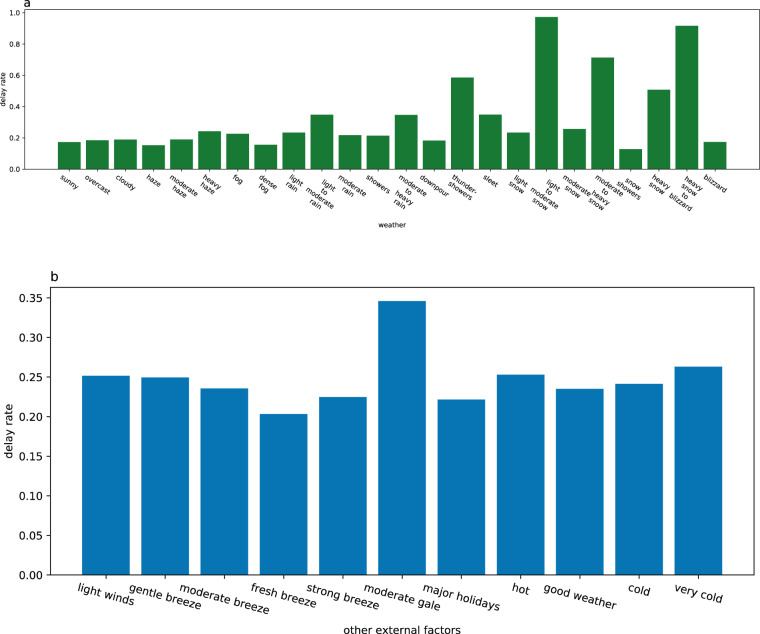
High-speed train delay rate under the influence of different external factors. (**a**) shows the relationship between various types of weather and delay rate. (**b**) shows the relationship between other external factors and delay rate.

We use the train delay rate under each external factor to quantify the relationship between an external factor and train operation, as shown in Fig. 8. (a) shows the relationship between weather and delay rate, (b) shows the relationship between other external factors and delay rate. The delay rate of the train increases significantly in typical bad weather such as light to moderate snow, heavy snow to Blizzard, mode to heavy snow, thunder towers and heavy snow, and the delay rate is more than 0.5, but there is no significant correlation with strong wind, holidays, high temperature and low temperature, and the delay rate is no more than 0.35.

#### The positive relationship between train delay and bad weather

**Fig. 9:**
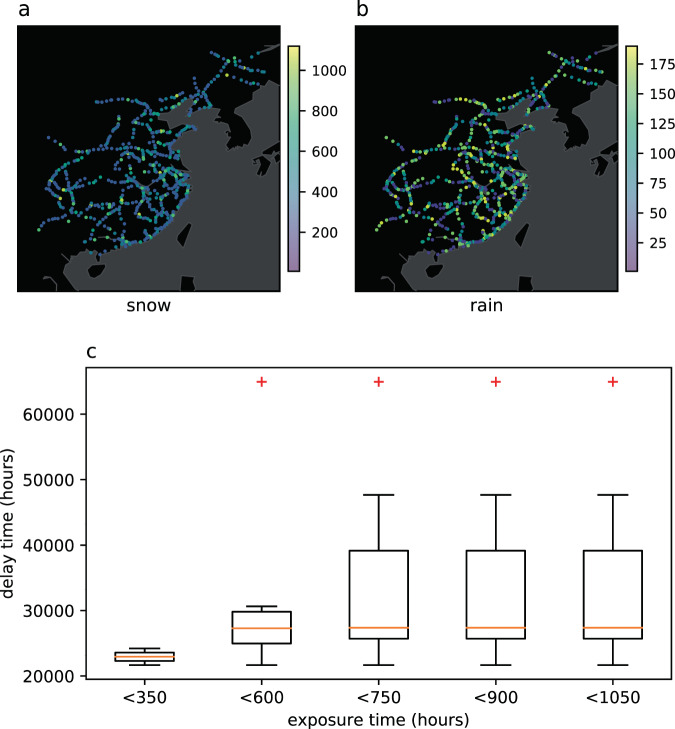
Relationship between train delay and exposure time in bad snow and rain weather. (**a**) and (**b**) shows the exposure time of bad rain and snow in the whole high-speed railway network. (**c**) shows the positive relationship between the exposure time of bad weather and the delay time.

Since the train operation data, station delay data and weather related data in our high-speed railway network dataset are collected in the same period, we can fully mining the relationship between train delay and bad weather. We visualize the delay time of each station exposed to snow and rain, as shown in Fig. 9(a,b). Most stations are exposed to snow for more than 200 hours, and most stations are exposed to rain for about 100 hours. In the 112 day data collected from the dataset, there is a good positive correlation between the total time of train exposure in bad weather and the specific delay time, as shown in Fig. 9(c).

#### Validation on power-law distribution characteristic of railway station community

In addition, we also verify the community structure of dynamic high-speed railway network. The community size of high-speed railway stations follows power-law distribution^4^:

1 $$power\_law(community\;size)=c\times {(communitysize)}^{-r}$$

where, $power\_law$ is a community power-law distribution function, and both *c* and *r* are constants greater than 0. Taking logarithms on the left and right sides of the equal sign in the above equation will get ${\rm{ln}}(power\_law)={\rm{ln}}c-r{\rm{ln}}(community\;size)$, that is, ${\rm{ln}}(power\_law)$ and ${\rm{ln}}(community\;size)$ meet the linear relationship. In double logarithmic coordinates, the power-law distribution will appear as a straight line with a negative slope of power exponent.

**Fig. 10:**
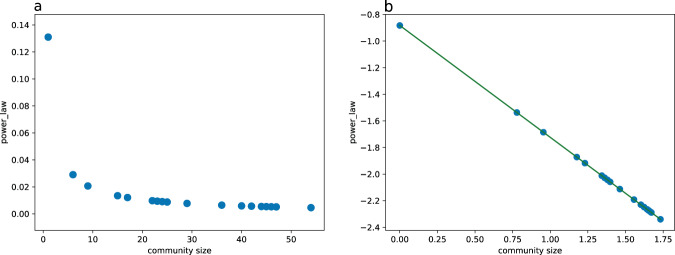
Power-law distribution of community size in high-speed railway network. (**a**) shows the station community size and power-law function before logarithm. (**b**) shows the station community size power-law distribution after logarithm. .

In order to validate this characteristic, based on the dynamic community structure in Fig. 5, we get the station community size in each time slice. Taking 09:00 to 12:00 am as an example, the relationship between the station community size before logarithm and the power–law function is shown in Fig. 10(a). Logarithm the size of the station community to obtain the relationship between the community size and the power-law function, as shown in Fig. 10(b), which follows the power-law distribution $power\_law(community\;size)=0.131\times {(communitysize)}^{-0.841}$.

After the validation from the above four aspects, we find the reliability of our dataset. The dataset can provide data support for the research on large-scale complex network, complex dynamical system, intelligent transportation, deep learning, data mining and other fields.

## Data Availability

We share our codes for data processing and generation in GitHub^31^. The detailed description of the codes is in the README.md.
